# The clinical correlation between Alzheimer's disease and epilepsy

**DOI:** 10.3389/fneur.2022.922535

**Published:** 2022-07-22

**Authors:** Dandan Zhang, Siyuan Chen, Shoucheng Xu, Jing Wu, Yuansu Zhuang, Wei Cao, Xiaopeng Chen, Xuezhong Li

**Affiliations:** Department of Neurology, Affiliated People's Hospital of Jiangsu University, Zhenjiang, China

**Keywords:** Alzheimer's disease, epilepsy, cognitive, temporal lobe epilepsy, antiepileptic drugs

## Abstract

Alzheimer's disease and epilepsy are common nervous system diseases in older adults, and their incidence rates tend to increase with age. Patients with mild cognitive impairment and Alzheimer's disease are more prone to have seizures. In patients older than 65 years, neurodegenerative conditions accounted for ~10% of all late-onset epilepsy cases, most of which are Alzheimer's disease. Epilepsy and seizure can occur in the early and late stages of Alzheimer's disease, leading to functional deterioration and behavioral alterations. Seizures promote amyloid-β and tau deposits, leading to neurodegenerative processes. Thus, there is a bi-directional association between Alzheimer's disease and epilepsy. Epilepsy is a risk factor for Alzheimer's disease and, in turn, Alzheimer's disease is an independent risk factor for developing epilepsy in old age. Many studies have evaluated the shared pathogenesis and clinical relevance of Alzheimer's disease and epilepsy. In this review, we discuss the clinical associations between Alzheimer's disease and epilepsy, including their incidence, clinical features, and electroencephalogram abnormalities. Clinical studies of the two disorders in recent years are summarized, and new antiepileptic drugs used for treating Alzheimer's disease are reviewed.

## Introduction

Alzheimer's disease (AD) and epilepsy are common nervous system diseases in older adults, and their incidence rates tend to increase with age. Epilepsy is approximately three times more common in individuals aged 65 years and older (1). Patients with mild cognitive impairment (MCI) and AD are more prone to have seizures (2). Specifically, the incidence of hippocampus-related degenerative diseases, such as AD and temporal lobe epilepsy (TLE), is increasing, partly because the hippocampus is susceptible to toxic substances such as excitatory amino acids, resulting in neuronal dysfunction, cognitive impairment, and death (3). Late-onset epilepsy is mostly secondary to brain diseases such as stroke, tumor, and neurodegenerative diseases, and many of them are of unknown etiology (4). In patients older than 65 years, neurodegenerative conditions accounted for ~10% of all late-onset epilepsy cases, most of which are AD (5). Epilepsy and seizure can occur in the early and late stages of AD, leading to functional deterioration and behavioral alterations (2). Evidence from both experimental and human studies has proven the existence of an association between epilepsy and AD (6). Transgenic mouse models with simulate familial AD can develop spontaneous epilepsy (7). Furthermore, seizures can lead to cognitive deficits, the trajectory of cognitive changes in patients with TLE differs from the pattern observed in healthy aging. Seizures also promote amyloid-β and tau deposits, leading to neurodegenerative processes (8). Thus, there is a bi-directional association between AD and epilepsy. Epilepsy is a risk factor for AD and, in turn, AD is an independent risk factor for developing epilepsy in old age (9). Many studies have evaluated the shared pathogenesis and clinical relevance of AD and epilepsy (5, 10, 11). In this review, we discuss the clinical associations between AD and epilepsy, including their incidence, clinical features, and electroencephalogram (EEG) abnormalities. Clinical studies of the two disorders in recent years are summarized (Table 1), and new antiepileptic drugs used for treating AD are reviewed.

**Table 1:** Summary of clinical studies of AD and epilepsy.

**Reference**	**Study type**	**Sample size (n)**	**Age (years)**	**Epilepsy type or seizure form**	**Follow up time**	**Results**	**Study limitations**
Lam et al. (12)	A cross-sectional study of epileptiform abnormalities in patients with AD	99	50–90	Epileptiform discharge	No	Epileptiform abnormalities occurred in 53% of patients with early-stage AD plus late-onset epilepsy related to AD	The sample size was small, and the sample had selection bias
Horvath et al. (13)	A prospective study of subclinical epileptiform activity accelerating the progression of AD	72	60–84	Epileptiform discharge	3 years	Subclinical epileptiform discharges were recorded in 54% of AD patients	EEG was not performed at the end of follow-up
Horvath et al. (14)	A retrospective and prospective study on the prevalence of epilepsy in AD using ambulatory EEG	42	70–84	Focal seizures	No	Seizures confirmed by EEG accounted for 24%	The sample size was small, and there was no distinction between early-onset and late-onset AD
Arnaldi et al. (15)	To retrospectively study the prevalence of epilepsy in AD	1,645	60–81	Generalized seizures, Focal seizures	1 year	The prevalence of epilepsy was 1.82% for AD	Retrospective data collection, lack of biomarkers for brain amyloidosis and tau protein
Difrancesco et al. (11)	A retrospective, observational, single center study on the prevalence of epilepsy in adults with presymptomatic AD	23	55–82	Generalized seizures, Focal seizures	No	In patients with AD, the prevalence of epilepsy before cognitive decline was 17.1 times that of the reference population	Retrospective data collection, samples were selected from a single center
Tedrus et al. (16)	Clinical study evaluating cognitive ability and its correlation with QEEG in patients with epilepsy	120	32–59	No	No	Adult patients with epilepsy had cognitive impairment that was significantly correlated with QEEG	Small sample size and single sample source
Voglein et al. (17)	A prospective study on the recurrence rate of epilepsy in AD	20,745	60–85	No	7.5 months	In AD patients, the risk of recurrence of seizures within 7.5 months was 70.4%	The influence of antiepileptic drugs on epilepsy recurrence was not analyzed, and the withdrawal rate was about 56%
Reyes et al. (18)	A cross-sectional study describing the nature and prevalence of cognitive impairment in TLE	217	55–80	Senile TLE	No	60% of older adults with TLE also had MCI	There was no longitudinal data or information on AD progression, and the sample representation was insufficient
Costa et al. (19)	A retrospective study on the risk stratification of cognitive decline in LOEU	24	59–73	LOEU	5.1years	During the follow-up of more than 5.1 years, 4 of 24 patients with LOEU developed AD	The sample size was small, and AD could not be accurately diagnosed according to stratification
Schnier et al. (20)	A nationwide cohort study of epilepsy and dementia events	563,151	>60	No classification	10 years	The risk of AD in patients with epilepsy was 1.6 times higher than that in the control group	Misclassification bias may exist from the case definition for epilepsy
Liguori et al. (21)	Prospective and observational study on cognitive performance of patients with LOEU	58	50–75	LOEU	1 year	MMSE and memory scores decreased significantly in LOEU at follow-up (12 months later)	The sample size was small

## Mechanistic evidences for co-morbidity of AD and epilepsy

The hippocampus shows similar pathological changes in patients with AD and individuals with epilepsy, such as loss of granulosa cells in the dentate gyrus, circuit reorganization, and damage to hippocampal neurons (22). The main pathological features of AD are amyloid β (Aβ) deposition outside neural cells and accumulation of hyperphosphorylated tau-protein in neurofibrillary tangles (23). These pathologies have also been observed in patients with TLE (24). In a study of 101 patients with TLE (aged 30–61 years) who underwent temporal lobectomy, 10 patients had Aβ plaques in brain samples (25). Tai et al. (26) performed pathological examinations on tissues from 33 patients aged 50–65 years who underwent temporal lobectomy for drug resistant TLE and found that 31 of the 33 patients (94%) had hyperphosphorylated tau. Hyperexcitability of nervous tissue is primarily responsible for increased seizure (27). Network hyperexcitability which induced by intracellular Aβ oligomers can occur in the early stages of AD and contribute to cognitive decline (28, 29). Recent studies have shown that presenilin 2 (PSEN2) knockout mice demonstrated early-life reductions in seizure threshold, and patients with PSEN gene variants also reported seizures (30). Furthermore, the increased tau pathology was associated with a greater decline in language learning, recall, and graded naming test scores (31). In patients with epilepsy, neurodegeneration occurs in the hippocampus, the temporal cortex, and the amygdala, with hippocampal sclerosis being the most characteristic structural change (32). Hippocampal sclerosis can cause neuronal loss and disruption of the balance between excitatory and inhibitory signaling in the sclerotic area, resulting in highly synchronous abnormal neuronal discharge (33). The hippocampus is an important part of the limbic system that plays a central role in memory function and is a site of early neurofibrillary tangle development (34). Therefore, pathological changes in the hippocampus may represent a link between AD and epilepsy.

**Figure 1:**
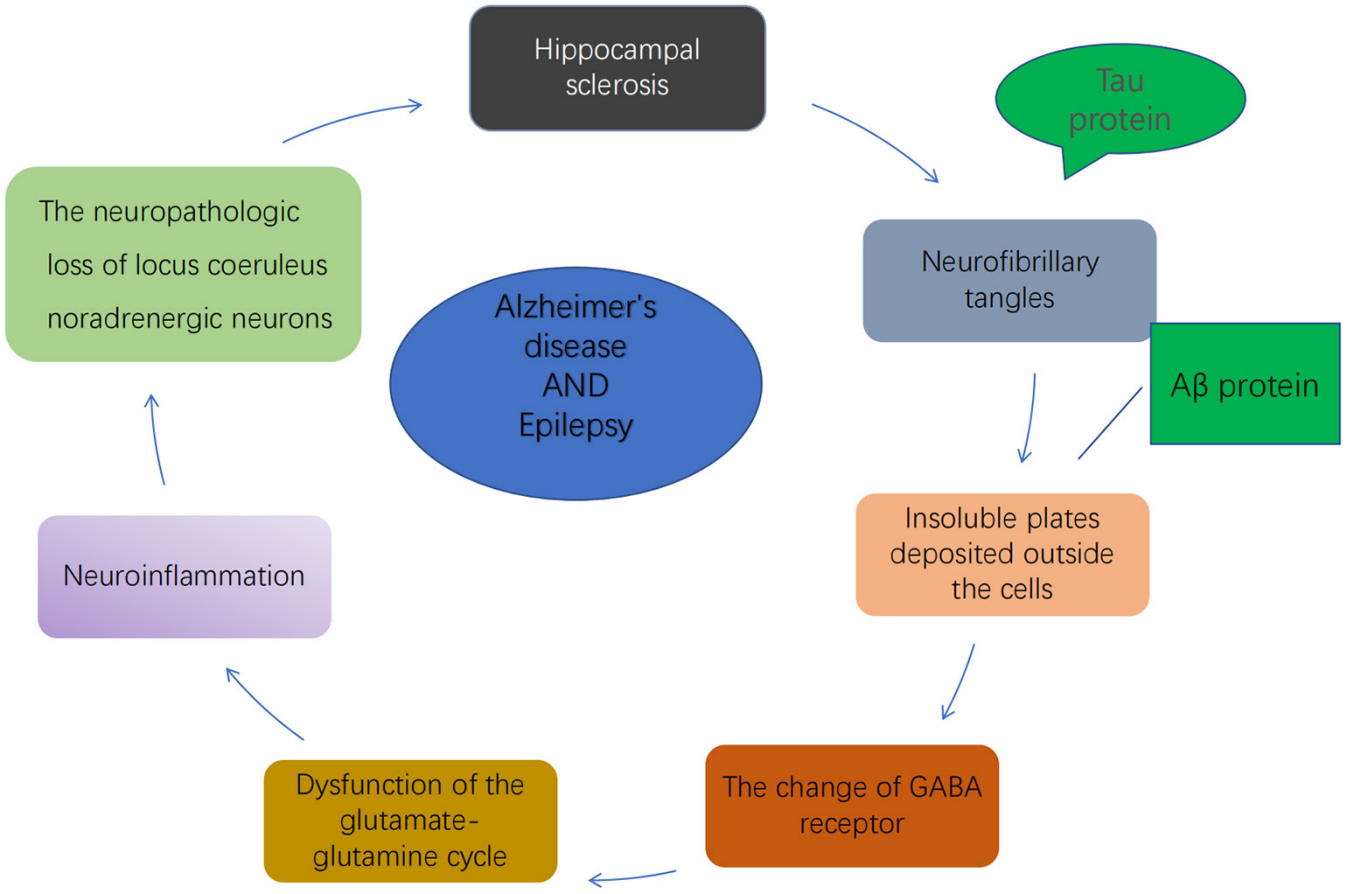
Possible mechanisms of AD and epilepsy association.

Other neurochemical changes associated with both AD and epilepsy include GABAergic (35) and glutamatergic alterations (36), Aβ and tau protein deposition (37), neuroinflammation (10), and damage to the noradrenergic nervous system in the locus coeruleus (38) (Figure 1).

## Clinical evidence of AD and epilepsy co-morbidity

AD is an important cause of senile epilepsy. Increased AD duration is associated with a higher risk of epilepsy (39). In addition, the risk of epilepsy in patients with AD is 10 times higher than that of individuals without AD in people over the age of 65 years (40). Horvath et al. (13) conducted a 3-year prospective follow-up study that included yearly cognitive assessments on 38 patients with AD and 20 healthy individuals and found that subclinical epileptiform discharges were recorded more frequently in patients with AD (54%) than in healthy, elderly (25%) individuals. Subclinical epileptiform activity was also observed in patients with amnestic mild cognitive impairment (aMCI) or early AD (28). Patients with familial early-onset AD have a higher risk of having seizures (41). Haoudy et al. (42) studied 25 patients with early onset AD and positive cerebrospinal fluid AD biomarkers. Based on epilepsy expert consultation and EEG examination, the results showed that 10 (40%) of these patients had epilepsy.

Epilepsy may play a causal role in AD development. In some cases, seizures may begin concurrently with onset of cognitive decline or even precede onset of AD (28). In AD patients, increased susceptibility to seizures and silent epileptiform activity due to disruption of the excitatory /inhibitory balance occur much earlier than cognitive impairment. The increase of epileptiform activity may be the main pathology in the early stage, which directly contributes to cognitive impairment (43). In a postmortem study (44), chronic epilepsy was associated with increased tau neurofibrillary tangles at mid-braak stages in patients aged 40–65 years. AD is a chronic, progressive disease that includes MCI and aMCI. Because the typical symptoms of AD may occur only after repeated seizures for several years, increasing numbers of studies have evaluated cognition in epilepsy. In one study (19), 13 out of 24 patients with late onset epilepsy of unknown etiology (LOEU) who were followed for 5.1 years had MCI. Most LOEU patients had an MCI status during seizures (59%) (45). The use of various antiepileptic drugs in the successful treatment of cognitive impairment in patients with AD provides an additional link between epilepsy and AD (46).

## Cognitive impairment in epilepsy

### Prevalence of cognitive impairment in epilepsy

In the UK, the prevalence of AD among patients with epilepsy is about 8% (47). In a population-based prospective study of seizures in adults aged > 17 years, Forsgren et al. (48) collected data of 563 patients with possible seizures in 34 months and found that seven cases were caused by AD (incidence 4%). In a cohort of 177 patients with newly diagnosed clinically with probable AD, 12 (6.8%) had a history of epilepsy and/or were using antiepileptic drugs at the time of diagnosis (49). Studies (50) have shown that 11% of adult patients with seizures suffer from AD. In a 10-year follow-up study, the 10-year cumulative incidence of dementia (including AD, vascular dementia) in patients with LOEU was 22% (51). To assess the relationship between epilepsy and dementia (including AD, vascular dementia) risk in Taiwan, Tsai et al. (52) conducted a retrospective cohort study that included 675 patients (age, ≥50 years) with epilepsy and 2,025 age-matched control subjects. Their results showed that individuals in the epilepsy cohort were at significantly increased risk of dementia compared with individuals in the control cohort. The risk of dementia in patients with epilepsy was also evaluated in the Framingham Heart Study (53). The study included follow-up information for 4,906 participants with epilepsy over the age of 65 years, and identified 51 cases of sporadic dementia (84% with AD type) in 43 patients with epilepsy and 129 controls without epilepsy. A study (54) of 247 untreated patients with newly diagnosed epilepsy showed that 49.4% of patients had impaired attention and executive function, and 47.8% had memory impairment.

### Clinical characteristics of cognitive impairment in epilepsy

Patients with epilepsy, especially late-onset epilepsy, often have cognitive impairment. Most seizure-related cognitive problems are affected by a variety of interrelated factors, including early age of seizure onset, seizure frequency, intensity and duration, and antiepileptic drug treatment (55). For example, patients diagnosed with LOEU show a significant decline in cognitive ability, unrelated to antiepileptic drug treatment, 12 months after diagnosis (21). In a study that assessed 38 elderly adults with epilepsy and 29 healthy controls, a comprehensive neuropsychological battery showed that the group with epilepsy had lower Mini-Mental State Examination (MMSE) and Dementia Rating Scale (DRS) total scores, performed worse on the attention and memory subscales of the DRS, and had poorer composite domain scores for verbal memory, visual memory, executive function, language, and processing speed when compared with controls (56). Wang et al. (57) studied 257 adult patients with epilepsy and observed that the overall cognitive function of patients with epilepsy was lower than the reference range for the Montreal Cognitive Assessment (MoCA) and clinical memory scale. Reyes et al. (18) studied 71 patients with TLE, 77 patients with aMCI, and 69 healthy controls. They found that 43 patients with TLE (60%) met the TLE-MCI standard and showed obvious memory and language defects. Subgroup analysis by age of seizure onset revealed that 63% of patients with early seizure onset (<50 years old) and 56% of patients with late seizure onset (≥50 years old) met the TLE-MCI standard. Patients with TLE-MCI showed greater language defects, while patients with aMCI forgot information more rapidly. Taylor et al. (58) found that memory and psychomotor speed were the most affected areas of cognitive function in patients with epilepsy.

Memory, processing speed, and higher executive function are the cognitive functions most prone to change within 12 months in newly diagnosed patients with epilepsy (59). For examples, when patterns of cortical atrophy and cognitive impairment were analyzed in 73 elderly patients with TLE (>55 years), 79 patients with aMCI, and 70 healthy controls were studied (60), results showed that the elderly patients with TLE exhibited medial temporal lobe atrophy similar to that observed in patients with aMCI. The TLE and aMCI groups also showed significant memory and language impairment when compared to the healthy control group. The association between medial temporal lobe atrophy and cognitive impairment in elderly people with TLE and aMCI demonstrates the risk of cognitive impairment associated with epilepsy. Johnson et al. (61) studied the relationship between late-onset epilepsy (LOE) and changes in cognitive ability over a 25 year period and found that LOE was associated with rapid declines in cognition, verbal memory, executive function, and word fluency.

### EEG characteristics of cognitive impairment in epilepsy

Interictal epileptic discharge (IED) are the EEG signs of epilepsies between seizures (62). Some cognitive loss might evolve in some epilepsies and patients if many and long term (63). Interictal activity results in significant changes in cognitive function, especially learning and memory (64). In patients with long-term TLE, the occurrence of IED is strongly related to cognitive impairment, and the most obvious is frontal lobe function (65). Liu et al. (62) performed EEG examinations and global cognitive assessments on 167 adult patients with and without epilepsy and showed that verbal fluency and language scores were reduced in patients with IED. They also found that sleep-phase IEDs were associated with lower cognitive performance. Interictal spike, especially if frequent and extensive, can impair cognitive abilities through interference with waking learning and memory, as well as memory consolidation, during sleep (66).

Moreover, Ung et al. (67) found that interictal epileptic activity outside the left hemispheric seizure onset zone impacted memory encoding, recall, and retrieval, while those inside the seizure onset zone did not. Hippocampal interictal epileptiform activity disrupted memory maintenance and retrieval, but not encoding cognition in humans (68). Horak et al. (69) also collected data of 80 patients with epilepsy who participated in the delayed free recall task while undergoing intracranial EEG monitoring and found that IED in the inferior-temporal, medial-temporal, and parietal areas significantly affected memory.

Frequent seizures can reduce the power and frequency of theta waves (70). Quantitative EEG (QEEG) can be used to objectively evaluate the power of radio waves that reflect cognition. The association between epilepsy and QEEG was studied in 80 adult patients with epilepsy and 40 healthy controls. The results showed that adult patients with epilepsy had cognitive impairment that significantly correlated with QEEG changes. Thus, QEEG may aid in understanding the pathophysiology of epilepsy and serve as an early marker of the cognitive changes that occur in epilepsy (16). Further evidence of the association between QEEG and cognitive changes in epilepsy is provided by Elsherif et al. (71), who administered cognitive tests and performed QEEG evaluation of EEG markers (median frequency, peak frequency, and alpha-to-theta ratio) in 45 patients with TLE. Researchers showed a strong negative correlation between cognition in patients with TLE and measured EEG markers. Thus, QEEG of these three EEG markers may be used to identify early cognitive impairment in patients with epilepsy.

## Epilepsy and seizure in AD

### Prevalence of epilepsy and seizure in AD

Epidemiological data on epilepsy in AD is limited, likely because neurodegeneration begins a few years prior to the emergence of clinical symptoms, and epilepsy may have been present in the pre-symptomatic stage of AD (15). The estimated prevalence of epilepsy in patients with dementia (including AD, vascular dementia, Lewy body disease) is about 5% (72). However, a recent prospective cohort study (73) with large sample size, long follow-up, and carefully characterized evaluations estimated the overall incidence of seizures in AD at ~1 per 200 person-years of observation, suggesting relatively lower frequencies than those reported in other studies. Vossel et al. (74) found that 42.4% of patients with AD exhibited epileptic activity compared with 10.5% in the control group. Furthermore, the overall cognitive ability of patients with epileptic activity decreased faster than that in the control group. Zelano et al. (75) assessed the risk of epilepsy in patients with AD. The results showed that the risk of epilepsy at 5 and 10 years after onset of AD was 2.1% and 4.0%, while that in the control group was 0.8 and 1.6%, respectively. However, Mahamud et al. (76) estimated that the risk of epilepsy diagnosis in the 5 years following a first, unprovoked seizure in patients with AD was 32%, compared to 31% in the control group. Cognitively asymptomatic individuals who harbor pathogenic autosomal dominant AD mutations were more likely to have seizures (77).

### Clinical characteristics of seizure in AD

Seizures may be particularly difficult to diagnose in patients with AD, as most reports of seizure symptoms in AD patients are provided by caregivers. It may be difficult for caregivers to describe seizure symptoms and to distinguish these symptoms from common manifestations of AD (such as hallucinations and delusions) (40). TLE may be an early feature of sporadic AD (78). Among patients with aMCI or AD with epilepsy, complex partial seizures are most common, and more than half of these seizures are non-convulsive (28). A study (46) showed that generalized tonic-clonic seizures occurred in 15–40% of patients with advanced AD complicated with epilepsy, and 70% of seizures in these patients were focal. Horváth et al. (14) analyzed the characteristics of 18 seizures in 10 patients with AD and found that 11% of the seizures were generalized tonic-clonic, 72% were focal seizures with impaired awareness, and 55% were non-motor seizures. Haoudy et al. (42) investigated 40 patients with early onset AD (EOAD) and found that the most common seizure types were tonic-clonic (25%), typical temporal seizures (25%), myoclonic (25%), focal extra-temporal (8%), and other seizure types (17%). Subclinical epileptiform activity in patients with AD has also been associated with cognitive ability (79). Vossel et al. (74) found that patients with AD and subclinical epileptiform activity have a faster decline in overall cognitive ability, as evidenced by a decrease of 3.9 points per year in their MMSE score. This is compared to the 1.6 points per year decrease observed in patients with AD without epileptiform activity.

Costa et al. (80) showed that the incidence rate of late-onset cryptogenic epilepsy in patients with advanced AD was associated with higher concentrations of AD markers in CSF. They retrospectively selected and studied 13 patients who met the diagnostic criteria for MCI. All 13 patients also met the criteria for clinical diagnosis of epilepsy prior to MCI diagnosis. Interestingly, epilepsy appeared 4–7 years earlier than AD. This phenomenon was termed epileptic prodromal AD, and researchers suggested that epileptic prodromal AD is an epileptic variant of AD. Furthermore, they suggested that the spectrum of AD should be extended to include epilepsy variants. In summary, epilepsy should be used as a phenotypic marker of AD (78).

### EEG characteristics of seizure in AD

Focal seizures in AD may be difficult to identify and may not be detected by surface EEG electrodes that only detect cortical activity (81). In patients with AD, IED was usually detected in electrodes around the frontotemporal and temporal lobe brain regions (78). Vossel et al. (28) found temporal epileptiform abnormalities were the commonest finding, although frontal and generalized discharges were also seen. The results were consistent with Sarkis's study results (82).

Because non-convulsive seizures may be masked by cognitive impairment, detection of epileptic-like activity may require long-term EEG monitoring (83). Long-term EEG recording, including during sleep, may result in a higher diagnostic rate (84). In a preliminary study (85), EEG abnormalities during sleep were associated with cerebrospinal fluid biomarkers, particularly hyperphosphorylated tau protein levels, suggesting the presence of preclinical AD. Horvath et al. (14) recorded electric ictal patterns twice during a recording period in 2 of 10 patients with AD, and clinically identified seizures in 8 of the 10 patients with AD using ambulatory EEG. Yu et al. (86) summarized the results of several research groups and found that epileptiform discharges, especially those with the specific characteristics of frequent, small spikes, temporal intermittent rhythmic delta activities, and paroxysmal slow wave events recorded using long-term scalp EEG, provided sufficient sensitivity and specificity for detecting the epileptogenic nature of AD. Horvath et al. (13) found that epileptiform discharges were associated with lower memory scores. Lam et al. (12) found that epileptiform discharges tended to occur during periods of wakefulness and during rapid eye movement sleep (REM sleep). Frequent spikes were particularly associated with epileptiform EEG and may serve as markers of hyperexcitability in AD.

The sensitivity and daily distribution of epileptiform discharges are different during different periods throughout the day. For example, Horvath et al. (87) found that EEG epileptiform discharges were more likely to be recorded between 8:00 and 16:00, while 82% of epileptiform discharges in sleep occurred during non-REM sleep. Moreover, AD progression was related to the high recurrence of seizures. Voglein et al. (17) investigated 20,745 individuals from the National Alzheimer's Coordinating Center and found that the risk of epilepsy recurrence within the following 7.5-month period was 70.4% in patients with AD. Furthermore, seizure prevalence increased with increasing duration of AD.

## Treatment

The close relationship between epilepsy and AD indicates that both epilepsy and AD require active intervention. Antiepileptic drugs (AEDs) improve cognitive symptoms and have antiepileptic effects (88). Use of AEDs can directly inhibit seizures and hyperexcitability, which may reduce Aβ accumulation (89). However, because cognitive function is particularly susceptible to decline in patients with AD, the possible neurocognitive effects of these drugs must be considered when prescribing AEDs for AD (90). Use of AEDs may increase the risk of developing AD (91).

Elderly individuals are at higher risk for developing chronic diseases and often take a variety of oral medications. Thus, drug-drug interactions that alter drug metabolism and/or bioavailability must be considered when prescribing AEDs in elderly individuals. These types of drug-drug interactions may induce a variety of adverse effects that aggravate cognitive impairment (92). For example, benzodiazepines may enhance GABA secretion by astrocytes, resulting in an increased risk for developing AD (93, 94). Therefore, benzodiazepines are not recommended for treatment of seizures in AD patients. Valproic acid (VPA) should also be avoided, because it may lead to cognitive decline (95). A retrospective (96) analysis of ten antiepileptic drugs prescribed to elderly, showed that cognitive adverse efects are common in patients on topiramate (TPM) followed by those on zonisamide (ZNS) and gabapentin (GBP). A meta-analysis (97) reported that LTG was associated with a lower probability of seizure freedom than LEV and had a limited effect on cognition in older people with epilepsy. However, Liu et al. (98) performed a randomized controlled trial on drug intervention that indicated that LEV seemed to improve cognition, whereas phenobarbital and LTG could worsen cognition. Additional studies are needed to further characterize treatment of AD-related epilepsy with LTG.

LEV is a novel antiepileptic drug that has the ability to reverse cognitive impairment (99). Vossel et al. (100) studied the effect of LEV on cognitive ability in AD patients with and without epileptic activity and found that LEV improved spatial memory performance and executive ability in patients with AD with epileptic activity. This effect may be due to reduced AD-associated network hyperexcitability. Brivaracetam (BRV) is an antiepileptic drug that was approved by the FDA in 2013 for the treatment of partial seizures. Brodie et al. (101) followed up the tolerability, safety, and efficacy of BRV for adjunctive treatment of focal (partial-onset) seizures in patients aged ≥65 years. Thirty-two cases were included and the results showed that BRV was effective with no significant impact on cognition. In animal experiments, BRV reversed memory deficits in a mouse model of AD (102). A retrospective analysis of 71 patients with epilepsy (> 60 years old) who were treated with lacosamide (LCM) showed that 60% of patients continued to take LCM at their 12-month follow-up visit, and no patients discontinued taking LCM due to cognitive issues (103). Perampanel (PER) is a third-generation antiepileptic non-selective AMPA receptor antagonist used to treat patients over the age of 12 years who have epilepsy. Ahn et al. (104) recruited 17 patients with epilepsy and treated them with PER for 6 months. In this cohort, PER did not induce cognitive decline. Lattanzi et al. (105) reviewed and analyzed the adverse events experienced by elderly patients with epilepsy who took PER at 12 different Italian epilepsy centers. The results showed that the common adverse events in elderly individuals taking PER were dizziness, irritability, and drowsiness. Cognitive decline was not common. Rohracher et al. (106) conducted a critical narrative review on the use of new AEDs in elderly patients with epilepsy. The results showed that PER and LCM had the lowest interaction risk and were considered first-line drugs for epilepsy treatment in elderly patients. Other studies of these AEDs are summarized in Table 2.

**Table 2:** Effects of new antiepileptic drugs on cognition.

**References**	**Study type**	**Sample size (n)**	**Age (years)**	**Antiepileptic drugs**	**Follow up time**	**Results**	**Defects (limitations)**
Schoenberg et al. (107)	Effects of LEV on cognition, emotion, and balance in healthy, elderly individuals	20	65–80	LEV	5 weeks	LEV had no adverse effect on cognition or balance in the elderly with epilepsy	The study duration was relatively short, and the sample size was small
Howard et al. (108)	Clinical trial of 2 different doses of minocycline and placebo in patients with mild AD	544	68–83	Minocycline	24 months	Minocycline did not delay the progression of cognitive or functional impairment in patients with mild AD within 2 years	Biomarkers were not used to confirm the diagnosis of AD and subject compliance was poor
Li et al. (109)	Effect of LCM on cognitive function and mental status in patients with epilepsy	251	Not limited	LCM	Not reported	LCM had limited effects on cognitive and emotional states	Number of references was limited
Liguori et al. (110)	To compare the cognitive side effects of LCM and carbamazepine	16	50–68	LCM	3 months	LCM showed an increase in the EpiTrack score used to measure cognitive ability during follow-up	The sample size was very small, and the data was not obtained systematically
Lattanzi et al. (111)	Adjunctive BRV in older patients with focal seizures	1,029	≥65	BRV	12 months	BRV was efficacious, had good tolerability, and no unexpected safety signals emerged	Retrospective and network study
Leppik et al. (112)	Effects of PER in elderly patients with epilepsy	28	≥65	PER	23 weeks	Efficacy and adverse event rates were found to be consistent with the adult population	analysis of PER is limited by the size of the elderly subgroup
Sarkis et al. (103)	Tolerability of ZNS in elderly patients with seizures	39	≥60	ZNS	23 months	ZNS and LCM had similar retention rates, 4 discontinuations due to cognitive or behavioral side effects	Side effects were retrieved from the chart rather than reported systematically by the patients
Pohlmann-Eden et al. (113)	Comparative effectiveness of LEV, VPA and CBZ among elderly patients with epilepsy	308	≥60	LEV	52 weeks	LEV may be a suitable option for patients aged ≥ 60 years with epilepsy.	Results are exploratory, since subgroup analysis by age was not powered
Musaeus et al. (114)	LEV alters oscillatory connectivity in AD	12	50–90	LEV	3 weeks	Decreases in coherence in the delta band (1–3.99 Hz) suggested a beneficial effect of LEV for patients with AD	There were no significant changes in cognitive performance after this single dose administration

## Conclusion and future directions

Studies have shown that epilepsy and AD share a common underlying pathology. Seizures, especially generalized tonic-clonic and complex partial/non-convulsive ones are typically associated with a transitory cognitive impairment, even in case of incidental and single seizures. A thorough investigation of the risk and etiology of recurrent seizures is needed. Early identification of epilepsy in patients with AD and in the AD prodromal stage, through various diagnostic techniques (including EEG), is critical. Seizures related to AD may precede or coincide with the onset of the cognitive decline. Early identification of non-convulsive seizures and the recognition of epileptiform EEG activity is of crucial importance for patient outcome. The new generation of AEDs is more suitable for treatment of elderly patients with epilepsy and neurodegenerative diseases due to their superior pharmacokinetic characteristics. Timing of intervention is critical. Most research on hyperexcitability in AD has focused on Aβ and Tau, but other factors, such as presenilin 2 (PSEN2), may contribute to hyperexcitability in AD. Future studies should focus on multiple potential mechanisms of hyperactivity in AD, as this may lead to further characterization of the shared pathogenesis of AD and epilepsy and may allow for identification of novel therapies.

## Author contributions

JW and SX searched the literature. WC and YZ conducted literature review and analysis. DZ and SC wrote original draft. XC did review and editing. XL was responsible for supervision. All authors contributed to the article and approved the submitted version.

## Funding

This work was supported by the scientific research plan of the Affiliated People's Hospital of Jiangsu University (Y201933).

## Conflict of interest

The authors declare that the research was conducted in the absence of any commercial or financial relationships that could be construed as a potential conflict of interest.

## Publisher's note

All claims expressed in this article are solely those of the authors and do not necessarily represent those of their affiliated organizations, or those of the publisher, the editors and the reviewers. Any product that may be evaluated in this article, or claim that may be made by its manufacturer, is not guaranteed or endorsed by the publisher.
